# Treatment regimens for laryngeal and hypopharyngeal squamous cell carcinoma: a “real life” multicenter study of 2307 patients

**DOI:** 10.1007/s00405-024-08990-6

**Published:** 2024-10-22

**Authors:** Andreas Knopf, Manuel Christoph Ketterer, Thomas K. Hoffmann, Simon Laban, Alexander Berghaus, Martin Canis, Christian Jacobi, Jens Peter Klussmann, Wendelin Föringer, Roland Laszig, Jens Pfeiffer, Henning Bier

**Affiliations:** 1 Hals-Nasen-Ohrenklinik, Klinikum Rechts der Isar, Technische Universität München, Ismaninger Str. 22, 81675 Munich, Germany; 2 Klinik für Hals-Nasen-Ohrenheilkunde, Kopf- und Halschirurgie, Universitätsklinik Ulm, Frauensteige 12, 89075 Ulm, Germany; 3 Klinik und Poliklinik für Hals-Nasen-Ohrenheilkunde, Klinikum der Universität München, Marchioninistraße 15, 81377 Munich, Germany; 4 Zentrum für Hals-, Nasen- und Ohrenheilkunde, Universitätsklinikum Giessen, Klinikstrasse 33, 35392 Giessen, Germany; 5 Department of Otorhinolaryngology, Medical Center-University of Freiburg, Faculty of Medicine, University of Freiburg, Killianstraße 5, 79106 Freiburg, Germany; 6 Department of Otorhinolaryngology, Head and Neck Surgery, Medical Faculty, University of Cologne, Cologne, Germany

**Keywords:** Laryngeal cancer, Hypopharyngeal cancer, Treatment, Surgery, Radiotherapy

## Abstract

**Objective:**

This retrospective multicenter study aimed to evaluate surgical versus conservative treatment in patients with hypopharyngeal and laryngeal cancer under real world conditions.

**Methods:**

This study included 2307 patients diagnosed with hypopharyngeal or laryngeal squamous cell carcinoma (SCC) in five German tertiary head and neck centers between 01/2004 and 12/2014. Overall, 783 patients with advanced SCC consecutively underwent laryng(opharyng)ectomy (L(P)E). Patient chart data regarding age, sex, tumor location, TNM status, grading, indication for L(P)E, treatment modalities, R status, postoperative complications, and hospitalization time were analyzed. Patients with lacking data and incomplete staging and those who refused treatment or did not comply with the recommended treatment were excluded from survival analysis.

**Results:**

A slight but significant increase was observed in L(P)E, referring to an increasing rate of tumor recurrence. While T1/2N0M0 laryngeal and hypopharyngeal cancer patients showed comparable overall survival (OS) for surgical and conservative treatment, surgery showed significantly better OS in lymph node-positive individuals and locally advanced tumor stages. Tumor recurrence occurred in more than one-third of the cases. In particular, in early glottic cancer recurrence, L(P)E represents a curative and safe treatment option, whereas in supraglottic and hypopharyngeal cancer, L(P)E was associated with reduced survival rates. Notably, 36% of patients with supraglottic cancer and 59% of patients with hypopharyngeal cancer recurrence could only be treated with palliative care.

**Conclusion:**

Comparable survival rates were demonstrated for cT1/2N0M0 laryngeal and hypopharyngeal SCC compared with primary chemo-/radiotherapy and larynx-preserving surgery. Better OS was achieved after surgery in nodal-positive patients and in those with locally advanced disease. Tumor recurrence should be anticipated in up to 39% of cases. Glottic cancer recurrence can be successfully and safely treated with L(P)E, whereas OS is reduced in hypopharyngeal cancer and possibly in supraglottic cancer.

**Supplementary Information:**

The online version contains supplementary material available at 10.1007/s00405-024-08990-6.

## Introduction

Therapy for laryngeal and hypopharyngeal cancer should strike a balance between achieving sufficient therapeutic radicality for optimal recurrence-free survival (RFS) and overall survival (OS) outcomes and preserving or reconstructing functional structures [1]. Patients with limited local disease burden (T1/2 status) usually undergo larynx-preserving therapy, including larynx-preserving surgery (LpreS) or primary chemo-/radiotherapy (pC/RT). Organ preservation focuses on the prevention of total laryng(opharyng)ectomy (L(P)E) that is commonly recommended in T3–T4 laryngeal and hypopharyngeal cancer [1]. Wolf et al. [2] demonstrated that laryngeal preservation in advanced laryngeal cancer is possible with pC/RT without significantly diminished OS. However, 56% of the included patients underwent salvage LE. Other studies revealed that pC/RT is an appropriate therapy for stage III or IV glottic and supraglottic cancer [3, 4]. However, Sanabria et al. concluded that OS and RFS differ significantly among different trials and study cohorts, and evidence supports L(P)E as the treatment of choice for patients with T4 cancers, whereas pC/RT is an acceptable alternative for T3 cancers, provided that all resources for treatment administration, follow-up, and surgical salvage are available [5]. Despite intensive investigation, the best therapeutic regimen for laryngeal and hypopharyngeal cancer remains controversial and reflects the significant lack of prospective randomized trials that compare surgical and non-surgical treatment modalities. We conclude that the randomization of patients undergoing total L(P)E vs. pC/RT will not be feasible in the future.

However, L(P)E and pC/RT are well-established therapeutic options in locally advanced laryngeal and hypopharyngeal cancer, and several patients decide to undergo pC/RT to obtain larynx preservation. Interestingly, the annual incidence of laryngeal and hypopharyngeal cancer (~ 210,000 diagnoses worldwide) [6] has decreased over the last decades, however, in Germany, the rate of L(P)E has remained stable [7–9]. These contradictory results of decreasing incidence in tumor diagnoses, increasing attempts of laryngeal preservation, and stable rates of L(P)E in Germany indicate that larynx-preserving therapy fails in some patients. This prompted us to perform a large retrospective multicenter study of five German university medical care centers to comprehensively assess the oncological outcomes of all consecutively treated patients with hypopharyngeal and laryngeal cancer. We aimed to assess actual data apart from study protocols, show time trends in therapeutic regimens, analyze the oncological outcome of different tumor stages and treatment modalities, and evaluate survival parameters in patients with tumor recurrence after larynx-preserving therapy for T1/2 cancer.

## Methods

### Study cohort

The current study involved five tertiary university hospitals (four comprehensive cancer centers) and included patients who were treated between January 2004 and December 2014. The oncological outcomes after surgery or multimodal therapy and pC/RT were comprehensively assessed in 2307 consecutively treated patients with de novo hypopharyngeal and laryngeal SCC. Patients with prior diagnosis or treatment for another head and neck malignancy were excluded.

Moreover, clinical parameters, indications, treatment modalities, and time trends were analyzed in 783 L(P)E patients who were consecutively treated in the 11-year period, including patients with de novo carcinoma or recurrent disease.

Tumor samples were histologically reviewed by at least two experienced pathologists. Dysplasia, carcinoma in situ, and other histological subtypes, such as adenocarcinoma, were excluded from the study. Clinical parameters and survival data regarding age, sex, tumor location, TNM status, grading, indication for L(P)E, treatment modalities, R status, postoperative complications, hospitalization time, recurrence, and death/loss to follow-up were retrospectively collected. Patients with lacking data and incomplete staging and those who refused or with incomplete surgical and/or conservative treatment (C/RT) were excluded from survival analysis. Comorbidities with potential impact on hospitalization time, including lung embolism, cardiac infarction, cardiac arrhythmia, cerebrovascular event, hormone crisis, vessel blowout, and pharyngocutaneous fistula, were determined.

Primary CRT was recommended in cases of nodal positivity and for tumors classified as T3/4, as well as in hypopharyngeal and supra-/subglottic laryngeal cancer. Adjuvant RT usually recommended in patients with T3/4 status and/or nodal positivity as well as in R1 situation. Concomitant chemotherapy was indicated in cases of extracapsular spread and in R1 status when adjuvant RT was already recommended. In general, for patients over 70 years of age, concomitant chemotherapy was critically evaluated due to increased toxicity.

### Statistical analysis and ethics

Differences between the groups were analyzed using the Chi-square test and Fisher’s exact test for categorical variables and the unpaired student’s *t*-test for continuous variables. The main endpoints OS and RFS were assessed, measuring the time from treatment to death of any cause and recurrence. Survival rates and curves were calculated and illustrated using the Kaplan–Meier method and further analyzed using the log-rank test for univariate analysis. Variables that revealed prognostic factors or effect modifiers of outcomes were subsequently evaluated with proportional Cox regression for multivariate analysis. Time trends were analyzed using linear regression. *p* < 0.05 indicated statistical significance. Statistical analyses were performed using SPSS (SPSS Inc., Chicago, IL). This study was performed in line with the principles of the Declaration of Helsinki. The local ethical committees approved the study (Munich TU: 151/15; Munich LMU: 24-0232; Ulm 30/18; Giessen: 21/16; Freiburg: 273/15).

## Results

### Outcomes of the different treatment modalities

#### Supraglottic cancer

**Fig. 1:**
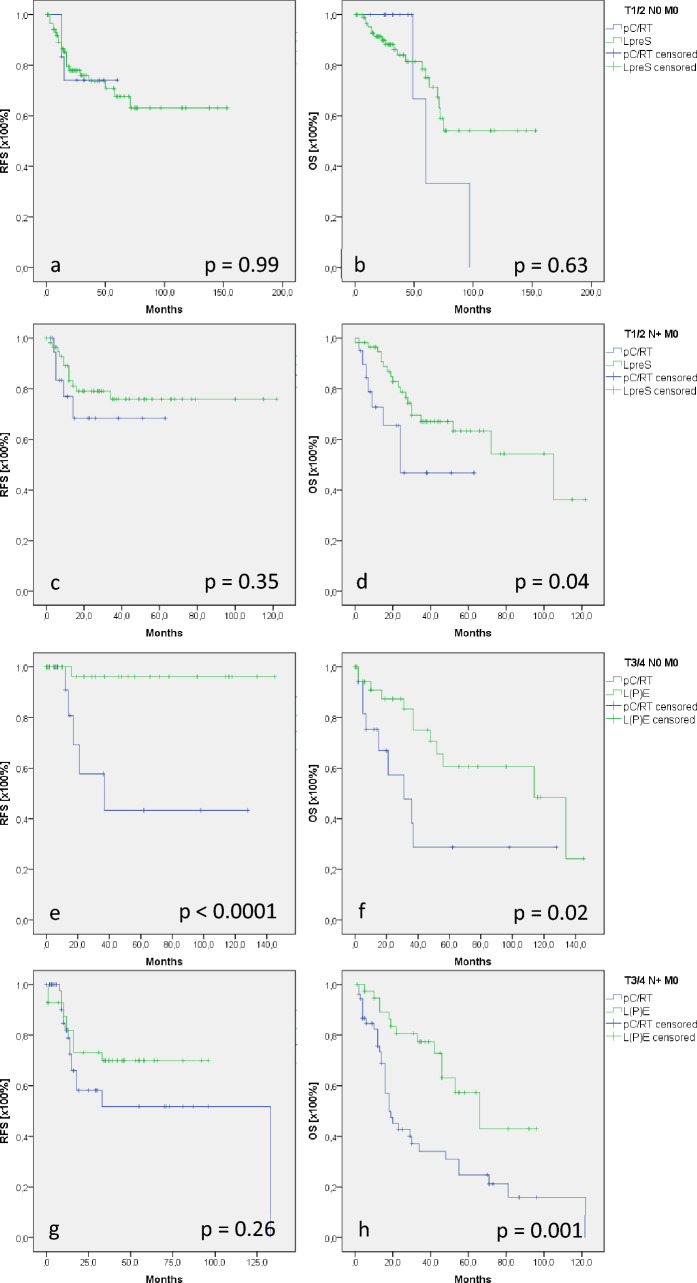
Recurrence-free and overall survival (RFS/OS) in supraglottic cancer. Patients with T1/2 N0 M0 supraglottic cancer revealed comparable RFS and OS comparing larynx-preserving surgery (LpreS) and primary chemo-/radiation (pC/RT) (**a**, **b**). Lymph node positivity in T1/2 supraglottic cancer resulted in comparable RFS (**c**) but significantly better OS (**d**) after LpreS than after primary pC/RT. In T3/4 N0 M0 supraglottic cancer, laryng(opharyng)ectomy (L(P)E) showed significantly better RFS/OS than pC/RT (**e**, **f**). Lymph node positivity in T3/4 supraglottic cancer resulted in comparable RFS (**g**) but significantly better OS (**h**) after L(P)E than after pC/RT

A substantial proportion of patients (67%) with supraglottic cancer underwent LpreS, including transoral approaches, lateral pharyngotomy, and vertical/horizontal partial laryngectomy (Table 1). Furthermore, 24% of patients were treated with primary pC/RT with curative intention (Table 1). Comparing patients with T1/2 N0 M0 supraglottic cancer who underwent LpreS and pC/RT, RFS (*p* = 0.99) and OS (*p* = 0.63) did not show differences between the groups (Fig. 1a, b). Subgroup analysis of patients who underwent larynx-preserving therapy revealed that patients who were conservatively treated were significantly older (mean age: 72 years) than those who were surgically treated (mean age: 60 years) (*p* < 0.0001). However, Cox regression excluded patient age as an OS-modifying parameter (*p* = 0.09; HR: 1.05; 95% CI [0.99; 1.11]).

**Table 1:** Distribution table of the study cohort, surgically and conservatively treated patients

	Supraglottic	Glottic	Subglottic	Hypopharynx	*p* Value
*n*	411	1009	28	859	
Age (years)	62	64	67	60	< 0.0001
Sex, *n* (%)					< 0.0001
Male	339 (83)	926 (92)	25 (89)	739 (86)	
Female	72 (17)	83 (8)	3 (11)	120 (14)	
T status, *n* (%)					< 0.0001
T1/2	198 (48)	737 (73)	9 (32)	300 (35)	
T3/4	213 (52)	272 (27)	19 (68)	559 (65)	
N status, *n* (%)					< 0.0001
N0	186 (45)	877 (87)	19 (68)	165 (19)	
Therapy, *n* (%)					0.009
Surgery	297 (72)	795 (79)	17 (61)	384 (45)	
LpreS	199 (67)	634 (80)	4 (24)	214 (56)	
L(P)E	98 (33)	161 (20)	13 (76)	170 (44)	
Neck dissection					< 0.0001
None	47 (16)	587 (74)	4 (24)	16 (4)	
Adjuvant treatment					< 0.0001
RT	138 (46)	107 (13)	7 (41)	137 (36)	
CRT	56 (19)	47 (6)	3 (18)	172 (45)	
Prim. conservative treatment	114 (28)	214 (21)	11 (39)	475 (55)	< 0.0001
RT	21 (18)	105 (49)	7 (64)	48 (10)	
CRT	77 (68)	104 (49)	4 (36)	339 (71)	
Palliative	16 (14)	5 (2)	0	88 (19)	

*L(P)E* laryng(opharyng)ectomy; *LpreS* larynx-preserving surgery

In contrast, survival parameters significantly differed in nodal-positive patients (T1/2 N + M0). Although no differences could be demonstrated in RFS (*p* = 0.35), patients who underwent LpreS showed a significantly better OS of 78 months compared with 37 months after pC/RT (*p* = 0.04, Fig. 1c, d). Consistent with lymph node-negative T1/2 patients, patients with N + status in the pC/RT group were significantly older than those who underwent LpreS (75 and 59 years, respectively; *p* < 0.0001, Supplement 1). Additionally, Cox regression excluded OS modification by patient age (*p* = 0.36; HR: 1.02; 95% CI [0.98; 1.06]).

In T3/4 N0 M0 supraglottic cancer, comparing pC/RT and L(P)E, there was significantly better survival in patients who underwent L(P)E. L(P)E patients showed a mean RFS/OS of 140/94 months, whereas pC/RT patients demonstrated a mean RFS/OS of 68/51 months (*p* < 0.0001; *p* = 0.02, Fig. 1e, f). The underlying cohort showed significant differences in sex distribution and tumor grading (Supplement 1). However, Cox regression for forward selection excluded sex and grading as OS-modifying parameters (sex: *p* = 0.64; HR: 0.74; 95% CI [0.21; 2.62]/grading: *p* = 0.41; HR: 1.51; 95% CI [0.38; 3.54]). After analyzing T3/4 N + patients, no differences were observed in RFS (*p* = 0.26; Fig. 1g); however, OS was significantly better after L(P)E (64 months) than after pC/RT (41 months; *p* = 0.001; Fig. 1h).

#### Glottic cancer

**Fig. 2:**
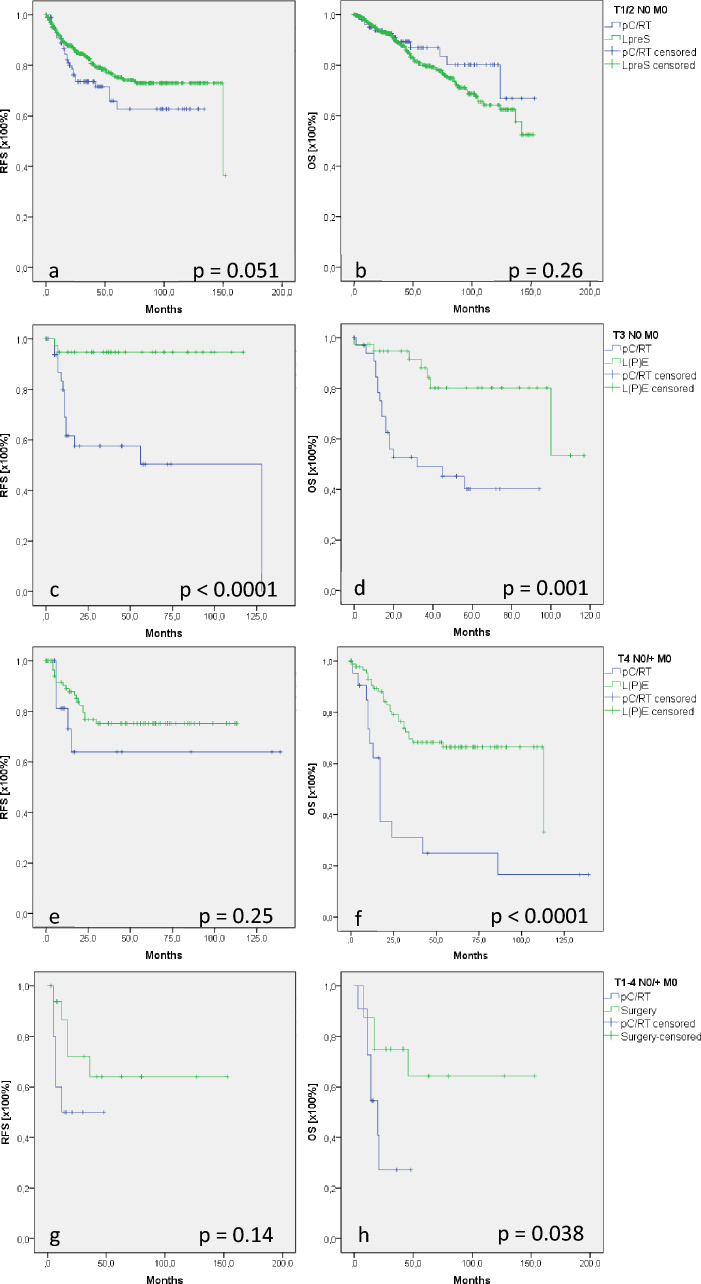
Recurrence-free and overall survival (RFS/OS) in glottis and subglottic cancer. RFS (**a**) and OS (**b**) did not show differences in patients with T1/2 N0M0 glottic cancer who underwent LpreS or pC/RT. In T3 N0 M0 glottic cancer, L(P)E patients demonstrated significantly better RFS/OS than pC/RT patients (**c**, **d**). Patients who suffered from T4 N0/ + M0 glottic cancer showed significant better OS after L(P)E than their conservatively treated counterparts; RFS was comparable in both groups (**e**, **f**). Subglottic cancer patients showed comparable RFS (**g**) but significantly better OS in surgically treated patients than in pR/CT patients (**h**)

In T1/2 N0 M0 glottic cancer, LpreS and pC/RT achieved comparable RFS (*p* = 0.051) and OS (*p* = 0.26; Fig. 2a, b). Treatment of T3 N0 M0 glottic cancer demonstrated significantly better RFS/OS in L(P)E patients (111/95 months) than in pC/RT patients (73/50 months; *p* < 0.0001/*p* = 0.001; Fig. 2c, d). The underlying cohort showed significantly older patients after pC/RT treatment compared with patients who underwent L(P)E (Supplement 2). Cox regression revealed significant disease modifying capability of patient’s age, even though the effects were small (*p* = 0.005; HR: 1.054; 95% CI [1.02; 1.09]). The comparison of advanced disease status (T4 N0/ + M0) demonstrated comparable RFS (*p* = 0.25; Fig. 2e), whereas OS was significantly better in L(P)E patients than in conservatively treated patients (83 vs. 42 months, *p* < 0.0001; Fig. 2f).

#### Subglottic cancer

The number of patients with curatively treated subglottic cancer was small (Table 1). Seventeen surgically treated patients demonstrated a comparable RFS (*p* = 0.14; Fig. 2g) to that of conservatively treated individuals. With a mean of 106 months, OS was significantly better in surgically treated patients than in pC/RT patients (24 months, *p* = 0.038; Fig. 2h).

#### Hypopharyngeal cancer

**Fig. 3:**
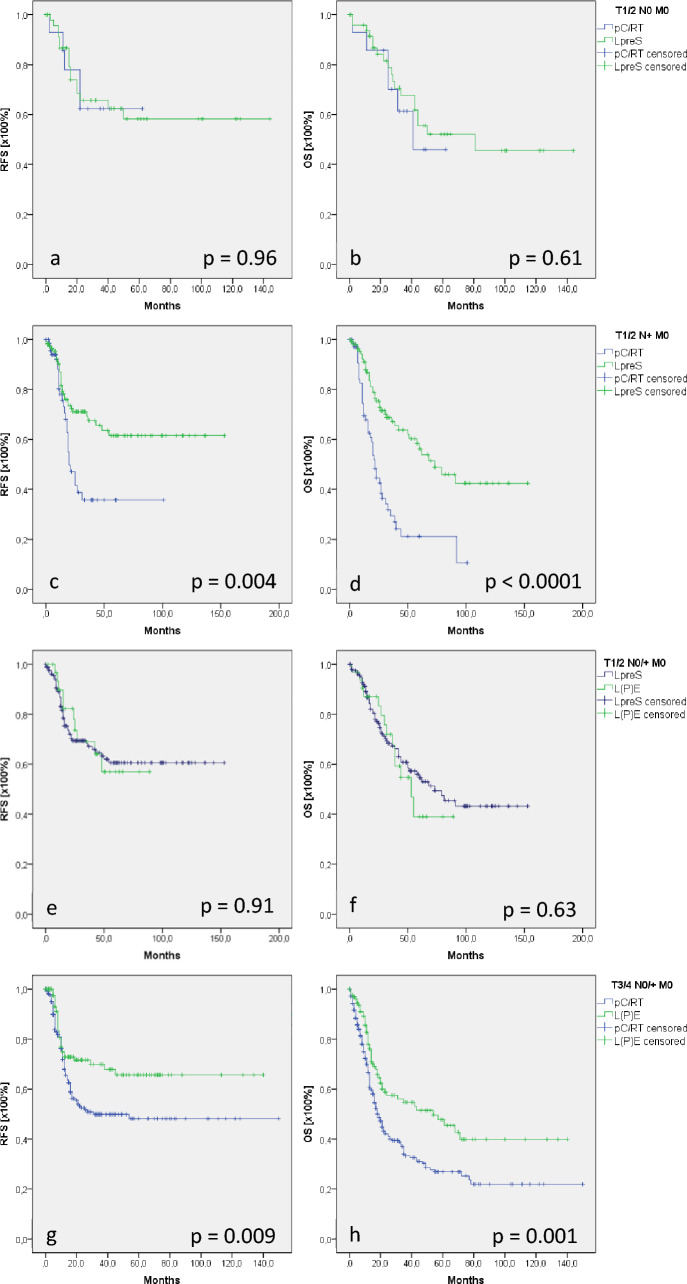
Recurrence-free and overall survival (RFS/OS) in hypopharyngeal cancer. RFS and OS did not show differences in patients with T1/2 N0 M0 glottic cancer who underwent LpreS or primary pC/RT (**a**, **b**). RFS and OS in T1/2 N + M0 (**c**, **d**) in hypopharyngeal cancer was significantly better in patients who underwent LpreS than in patients who had pC/RT. No differences in T1/2 N0/ + M0 staged cancer were observed comparing LpreS and L(P)E (**e**, **f**). In T3/4 N0/ + M0 hypopharyngeal cancer, OS/RFS was significantly better after L(P)E than after pC/RT (**g**, **h**)

Consistent with the results in supraglottic and glottic cancer, T1/2 N0 M0 hypopharyngeal cancer showed comparable RFS (*p* = 0.96) and OS (*p* = 0.61) in patients who underwent LpreS and pC/RT (Fig. 3a, b). As seen in supraglottic cancer, lymph node positivity in T1/2 hypopharyngeal cancer resulted in significantly better RFS/OS in patients after LpreS (102/87 months) than after pC/RT (47/36 months, *p* = 0.004/*p* < 0.0001; Fig. 3c, d). Interestingly, we could find significantly different OS or RFS comparing patients with T1/2 N0/ + M0 hypopharyngeal cancer who were treated with LpreS versus L(P)E (Fig. 3e, f). Differential analysis of the underlying cohort demonstrated significantly higher R status and less extended neck dissection in patients who underwent LpreS than those who underwent L(P)E (Supplement 3). However, cox regression for forward selection did not reveal any disease-modifying potential (R status: *p* = 0.11; HR: 1.20; 95% CI [0.96; 1.49]; neck dissection: *p* = 0.28; HR: 1.25; 95% CI [0.84; 1.86]. The comparison of L(P)E and pC/RT in T3/4 N0/ + M0 hypopharyngeal cancer revealed significantly better RFS (97 vs 79 months) and OS (71 vs 49 months) in L(P)E patients than in pC/RT patients (*p* = 0.009/*p* = 0.001; Fig. 3g, h).

### Treatment strategies for de novo laryngeal and hypopharyngeal cancer

In total, 2307 patients with de novo laryngeal and hypopharyngeal cancer were retrospectively analyzed. Supraglottic cancer was diagnosed in 411 patients (18%), glottic cancer in 1009 (44%), subglottic cancer in 28 (1%), and hypopharyngeal cancer in 859 (37%) (Table 1). Significant differences were found in age and sex distribution between primary tumor sites. Patients with hypopharyngeal cancer were significantly younger than those with laryngeal cancer (*p* < 0.0001), and in glottic cancer, a significantly higher proportion of male patients was observed when compared with other tumor locations (*p* < 0.0001; Table 1). TNM status at the time of diagnosis showed significant differences between the groups. The majority of patients with glottic cancer were diagnosed with T1/2 N0 M0 disease, and all other tumor locations showed T3/4 cancer in > 50% of patients (*p* < 0.0001; Table 1). In particular, hypopharyngeal and subglottic cancers were diagnosed with advanced T status (T3/4) in 65% and 68% of cases, respectively. Late diagnosis and early locoregional metastatic outgrowth were illustrated in lymph node positivity in > 80% of hypopharyngeal cancer patients. Interestingly, 68% of patients with subglottic cancer were diagnosed with N0 neck despite advanced T status. Lymph node negativity was diagnosed in 87% of patients with glottic cancer and 45% of patients with supraglottic cancer (*p* < 0.0001; Table 1). Distant metastastatic outgrowth at the time of diagnosis was observed in 1% of patients with glottic cancer and in 11% with hypopharyngeal cancer (*p* < 0.0001). The status of glottic cancer was highlighted after histological examination. Strikingly, the majority of tumor specimens in all primary sites were graded G2/3, but in patients with glottic cancer a higher prevalence of G1 cancer was observed (*p* < 0.0001, Table 1). Regarding subsequent TNM status, treatment modalities differed significantly between the tumor locations (*p* < 0.0001–0.009). Overall, 1865 patients (81%) underwent larynx-preserving therapy, with 814 patients undergoing pC/RT and 1051 undergoing LpreS. However, 109 newly diagnosed patients underwent C/RT with palliative intention (Table 1). In total, 442 patients (19%) were initially treated with L(P)E. The proportion of larynx-preserving therapy differed between primary tumor locations, ranging from 84% in glottic cancer, 80% in hypopharyngeal cancer, and 76% in supraglottic cancer to 54% in subglottic cancer (Table 1). Patients with supraglottic cancer were predominantly treated surgically (72%). LpreS was performed in almost two-thirds of the patients. Both L(P)E and LpreS were usually combined with neck dissection. Adjuvant treatment was mandatory in 64% of patients, comprising RT in 45% and C/RT in 19% of cases. In contrast, 795 surgically treated patients with glottic cancer underwent LpreS in 80% of cases. In these cases, neck dissection was not performed in 74% of patients and adjuvant treatment was avoided in 81% of cases. Subglottic cancer was treated with surgery (61%) or primary pC/RT (39%). Surgical approaches referred to L(P)E and neck dissection in 76% of patients and were supplemented with adjuvant C/RT in 59% of patients. Patients with hypopharyngeal cancer underwent surgical approaches in only 45% of cases. Interestingly, 56% of surgically treated patients underwent LpreS (Table 1).

### Clinical characterization and time trends in L(P)E at first pass or tumor recurrence

Between January 2004 and December 2014, a total of 783 patients underwent L(P)E due to head and neck SCC. L(P)E at first pass was performed in 80% of patients (*n* = 624), while 159 patients (20%) underwent L(P)E due to local tumor recurrence (Table 2). Subsequently, there was a strong tendency towards an older population in patients with tumor recurrence (*p* = 0.08; data not shown). While patients who were treated by L(P)E at first pass showed a more balanced distribution of primary tumor sites, there was a significant higher proportion of glottic cancer in the group of patients suffering from tumor recurrence (*p* < 0.001; Table 2). In both, L(P)E at first pass and in local tumor recurrence most patients demonstrated T3/4 status. However, patients with L(P)E at first pass showed a significant higher N-status when compared with patients in tumor recurrence that directly referred to a higher number of patients with glottic cancer and N0 status at the time of local tumor recurrence (*p* < 0.0001; Table 2). The shift towards a pronounced occurrence of glottic cancer in the group of patients with L(P)E in recurrent disease also resulted in a significant lower rate of adjuvant treatment when compared with patients who underwent L(P)E at first pass (*p* < 0.0001; Table 2).

**Table 2:** Comparison table of L(P)E at first pass and L(P)E in tumor recurrence

	L(P)E in rT +	L(P)E at first pass	*p*-Value
*n*	159	624	
Location, *n* (%)			< 0.0001
Supraglottic	27 (17)	136 (22)	
Glottic	94 (59)	193 (31)	
Subglottic	5 (3)	22 (4)	
Hypopharyngeal	23 (14)	171 (27)	
Oropharyngeal (vallecular)	9 (6)	100 (16)	
Other	1 (1)	2	
T status, *n* (%)			0.47
T1/2	32 (21)	78 (13)	
T3/4	127 (79)	546 (87)	
N status, *n* (%)			< 0.0001
N0	113 (71)	274 (44)	
N +	46 (29)	350 (56)	
Adjuvant therapy			< 0.0001
None	83 (52)	140 (22)	
RT	36 (23)	245 (39)	
CRT	40 (25)	239 (38)	

However, postoperative complications and mean hospitalization time (20 vs 21 days) were comparable in both groups, indicating that L(P)E is a safe surgical procedure (Supplement 4). Postoperative complications occurred in only 15% and 19% of patients demonstrating pharyngocutaneous fistula being the most frequent event, whereas cerebrovascular, cardiac, and pulmonary events occurred infrequently (Supplement 4).

Linear regression analysis and *t*-test revealed slight but significant increases over time in patient age (*R*^2^ = 0.02; *p* < 0.001), glottic cancer (*R*^2^ = 0.03; *p* = 0.024), and L(P)E because of local tumor recurrence (*R*^2^ = 0.01; *p* = 0.005; Supplement 4) between January 2004 and December 2014.

### Treatment strategies and outcomes after tumor recurrence in laryngeal and hypopharyngeal cancer

The proportion of recurrent disease (including all sites of tumor recurrence [rTNM]) ranged from 21% in glottic cancer to 39% in subglottic cancer. Local tumor recurrence was diagnosed in 12% of patients with supraglottic cancer, 18% with glottic cancer, 25% with subglottic cancer, and 14% with hypopharyngeal cancer. Locoregional lymph node involvement ranged from 6% in glottic cancer to 10% in hypopharyngeal cancer. Metachronous distant metastatic outgrowth was diagnosed in 4% of patients with glottic cancer, and in 18% of patients with subglottic or hypopharyngeal cancer alike. Interestingly, 51% of patients with supraglottic, 58% of patients with subglottic, and 74% of patients with glottic cancer recurrence showed T1/2 status at initial diagnosis. Conversely, 65% of patients with hypopharyngeal cancer recurrence were initially staged with T3/4 disease. Initial N0 status in patients with recurrent disease ranged from 26% in hypopharyngeal to 91% in glottic cancer.

**Fig. 4:**
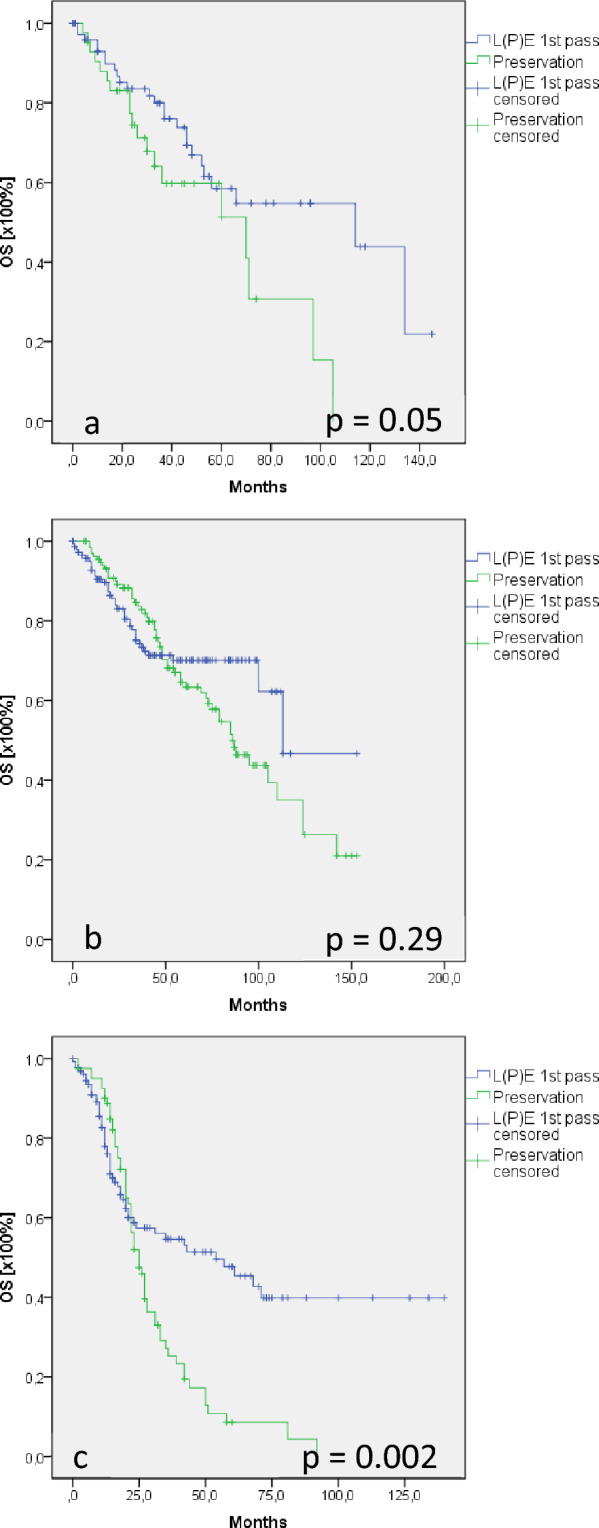
Overall survival (OS) in tumor recurrence after LpreS. Survival analysis compared patients who suffered from tumor recurrence after larynx preservation in T1/2 N0/ + M0 supraglottic and T1/2 N0 M0 glottic cancer with T3/4 N0/ + M0 staged patients who underwent L(P)E at first pass. OS was comparable in patients with supraglottic (**a**, *p* = 0.05) and glottic (**b**, *p* = 0.29) cancer and who were curatively treated for tumor recurrence and L(P)E patients at first pass. In contrast, OS was significantly worse in curatively treated patients with hypopharyngeal cancer (*p* = 0.002) when compared with patients treated with L(P)E at first pass (**c**)

In particular, in T1/2 N0/ + M0 supraglottic and hypopharyngeal cancer, surgical and conservative larynx preservation was frequently performed. In the cohort of patients with recurrent disease, subgroup analysis revealed that rT + status was diagnosed in 14–18% of T1/2 N0/ + M0 staged patients. Surgical and conservative larynx-preserving therapy is often recommended, arguing that further treatment options, particularly L(P)E, can be offered in cases of recurrence. This recommendation is inherently associated with a persisting curative treatment option, even after tumor recurrence. Therefore, we comprehensively analyzed the OS of patients who underwent L(P)E because of rT3/4 rN0/ + M0 tumor recurrence after initial larynx preservation (surgical or conservative) in T1/2 N0 M0 glottic and T1/2 N0/ + M0 supraglottic/hypopharyngeal cancers and compared this cohort with patients suffering from de novo T3/4 N0/ + M0 disease who underwent L(P)E at first pass. Patients with recurrent supraglottic cancer after LpreS (*n* = 42) showed a mean OS of 59 months, whereas patients with T3/4 N0/ + M0 and L(P)E at first pass (*n* = 83) demonstrated 89 months OS (*p* = 0.05, Fig. 4a). In glottic cancer, no differences were found between the groups. While 150 patients with L(P)E at first pass showed a mean OS of 104 months, 138 patients with recurrent disease demonstrated a mean OS of 89 months (*p* = 0.29; Fig. 4b). In contrast, 130 patients with T3/4 N0/ + M0 hypopharyngeal cancer and L(P)E at first pass showed significantly better survival (71 months) than 81 patients with tumor recurrence (31 months) (*p* = 0.002; Fig. 4c).

Moreover, 87% of patients with tumor recurrence after T1/2 N0 M0 glottic cancer were curatively treated, whereas the number of patients with supraglottic or hypopharyngeal cancer who were treated with palliative intent for recurrent disease was significantly higher (36% and 59%, respectively).

## Discussion

Therapeutic regimens for hypopharyngeal and laryngeal cancer still are a matter of great debate among head and neck cancer specialists. The lack of prospective randomized trials, limited comparability between different studies, and the importance of functional aspects in the decision-making highlight the complexity of the subject. While a significant increase in randomized trials comparing surgical approaches vs. C/RT cannot be expected owing to patient compliance, real-world data, as presented in the current study, help to reflect on contemporary treatment regimens.

Until 1991, L(P)E and adjuvant RT were the only therapeutic choices for advanced laryngeal and hypopharyngeal cancer patients. Wolf et al. [2] and the Department of Veterans Affairs laryngeal cancer study group showed that primary C/RT led to complete tumor response in 31% and partial response in 54% of cancer patients. Nevertheless, 36% of these patients underwent “salvage” L(P)E after pC/RT. Following studies comprehensively analyzed different conservative larynx-preserving approaches and concluded that radiotherapy with concurrent administration of cisplatin is superior to induction chemotherapy followed by radiotherapy or radiotherapy alone in patients with laryngeal cancer [3, 10]. Previous studies have shown that low-volume cT4a laryngeal cancer or less has no diminished OS with pC/RT compared with L(P)E treatment [2, 3].

Currently, induction therapy represents more of a clinico-biological biomarker to decide between complete CRT or salvage L(P)E than being part of a fixed conservative approach [11–14].

In a comprehensive review of advanced laryngeal cancer, Sanabria et al. [5] concluded that L(P)E is the treatment of choice in T4 SCC patients. These results agree with our study, which demonstrated significantly better OS in patients with T3/4 N0/ + M0 supraglottic cancer, T4 N0/ + M0 glottic cancer, and T3/4 N0/ + M0 hypopharyngeal cancer who had L(P)E than in those who underwent pC/RT. Additionally, Sanabria et al. concluded that pC/RT may be an acceptable alternative for laryngeal T3 cancer patients when treatment, follow-up, and surgical “salvage” are available and possible [5]. This reflects the heterogeneity of the results in T3 laryngeal cancer patients. In our cohort, patients with T3/4 N0 M0 supraglottic cancer, particularly patients with T3 N0 M0 glottic cancer, showed significantly better RFI and OS after L(P)E than their conservatively treated counterparts. These results disagree with those of Ko et al. [4] who demonstrated comparable results for T3 N0 M0 glottic cancer. These contradictory results may refer to a more aggressive therapeutic approach in our cohort, including L(P)E and a higher proportion of adjuvant C/RT. Moreover, D'Ascanio et al. [15] highlighted that Ko et al. [4] did not categorize their patients into subgroups. Glottic T3 carcinoma can lead to different outcomes following pC/RT depending on the anatomically sickened region (vocal cord with fixation vs. paraglottic space, minor thyroid cartilage erosion, etc.). Recent data revealed that after careful patient selection, pC/RT can avoid L(P)E, which is associated with reduced quality of life [4, 5]. In this scenario, induction-CT as a clinico-biological marker to decide whether to continue C/RT or perform salvage L(P)E may have a major clinical impact [10–12, 16, 17]. However, laryngeal preservation after pC/RT does not necessarily result in functional preservation. Particularly, hypopharyngeal and laryngeal cancer, increased patient age, and advanced T status were identified as independent risk factors for late toxicity, such as laryngeal dysfunction, swallowing dysfunction, long-term dysphagia, and dependence on a feeding tube for > 2 years following pC/RT, resulting in decreased quality of life [18–21]. More recently, studies demonstrated that L(P)E without adjuvant therapy is sufficient in pT3 pN0 cM0 R0 glottis cancer [22] and avoids adjuvant treatment. The ideal treatment recommendation for T3 N0 M0 glottic cancer is controversial.

Although the final treatment recommendation for T3 N0 laryngeal cancer remains unclear, T1/2 N0 supra-/glottic cancer shows a promising overall survival (OS) rate of 82–100%, with no significant differences between treatment modalities [23–29]. Our study demonstrated comparable OS for LpreS and pC/RT in T1/2 N0 supraglottic, glottic, and hypopharyngeal cancer. Interestingly, nodal positivity resulted in significantly better OS for T1/2 N + supraglottic and hypopharyngeal cancer after LpreS than after pC/RT. However, laryngeal preservation in both surgical and conservative regimens should anticipate tumor recurrence and its subsequent management. These patients often undergo L(P)E [2].

Wei [30] highlighted that optimal treatment for hypopharyngeal cancer requires the surgeon to consider the pathological behavior, tumor extension, and cervical lymph node status. In agreement with Wei surgical and conservative approaches yield comparable outcomes in early-staged (T1/2 N0 M0) hypopharyngeal carcinoma. However, for advanced disease, radical surgery followed by adjuvant C/RT might be favorable. Based on Wei's findings [30], it is essential to consider submucosal extension with perilesional infiltration, particularly in cases with a higher R-status in LPreS. Accordingly, our study demonstrated that hypopharyngeal cancer patients underwent more often adjuvant CRT, most likely due to efforts of laryngeal preservation, which involved accepting close margins and a higher R-status. Submucosal tumor extension and limited options of functional larynx reconstruction reflects that substantial proportion of our underwent L(P)E as primary surgical therapy.

Concordant with a previous study, our study demonstrated an increase in L(P)E in tumor recurrence [31]. This subsequently addresses how many patients undergo L(P)E because of tumor recurrence and if these patients suffer from reduced OS. The current study included 783 L(P)E patients. L(P)E at first pass was performed in 80% of patients (*n* = 624), whereas 159 patients (20%) underwent L(P)E because of local tumor recurrence. While patients who were treated by L(P)E at first pass showed a balanced distribution of primary tumor locations, a significantly higher proportion of glottic cancer was noted in patients with tumor recurrence. The recurrence rate of the included patients was not significantly different between the different treatment modalities. To address whether OS is not diminished by tumor recurrence, we analyzed patients who underwent curative L(P)E because of rT3/4 rN0/ + M0 tumor recurrence after initial larynx preservation (surgical and conservative) in T1/2 N0 M0 glottic and T1/2 N0/ + M0 supraglottic/hypopharyngeal cancers and compared this cohort with patients suffering from de novo T3/4 N0/ + M0 disease who underwent L(P)E at first pass. In hypopharyngeal cancer, a significantly better survival was noted in patients who underwent L(P)E at first pass compared with patients who underwent L(P)E because of rT3/4 rN0/ + M0 disease. Additionally, a better OS was noted in supraglottic cancer patients after L(P)E at first pass. In glottic cancer, no differences between the groups were observed. Notably, 87% of patients with tumor recurrence after T1/2 N0 M0 glottic cancer were curatively treated, whereas the number of patients with supraglottic and hypopharyngeal cancer who were treated with palliative intention increased. These results do not suggest L(P)E in T1/2 supraglottic or hypopharyngeal cancer,however, preoperative discussion should be adapted because L(P)E in tumor recurrence is not associated with the same OS as salvage L(P)E. This result should be highlighted because the term “salvage laryngectomy” is used incorrectly in several recent studies. Tupper [32] and Sanabria et al. [33] defined surgical salvage as part of primary tumor treatment when initial conservative treatment lacks complete response. Further, semantic inaccuracy deteriorates the comparability of contemporary studies.

One limitation of this study is its retrospective nature, which may introduce potential data bias. Additionally, five different centers were involved in data collection, potentially leading to variations in surgical approaches and interpretations of the need for therapy. Performance status and functional data were not evaluated due to the retrospective design and the absence of relevant records and examinations. In addition, TNM classification was done using the UICC 7th edition. Histological examination misses detailed assessment of nodal extracapsular spread (ECS + /N3b status) and transfer to the 8th edition might be delusive at this point. Although recommendation of adjuvant CRT did not change over the time, the underlying reason (R1 vs. ECS +) can`t be assessed in a substantial proportion of patients.

## Conclusion

Therefore, this study demonstrated comparable survival rates for cT1/2N0M0 laryngeal and hypopharyngeal SCC compared with pC/RT and LpreS. Better OS was achieved after surgery in nodal-positive patients and in those with locally advanced disease. Regarding recurrence rates of up to 39% after larynx-persevering therapy, glottic cancer can be successfully and safely treated with L(P)E, whereas OS is reduced in hypopharyngeal and possibly in supraglottic cancer.

## Supplementary Information

Below is the link to the electronic supplementary material.

Supplementary file1 (DOCX 45 KB)

## Abbreviations

SCC: Squamous cell carcinoma
C/RT: Chemo-/radiotherapy
OS: Overall survival
ORL: Otorhinolaryngology
LE: Laryngectomy
L(P)E: Laryng(opharyng)ectomy
RT: Radiotherapy
RFS: Recurrence-free survival
LpreS: Larynx-preserving surgery

## References

[1] Jones TM, De M, Foran B, Harrington K, Mortimore S (2016) Laryngeal cancer: United Kingdom National multidisciplinary guidelines. J Laryngol Otol 130(S2):S75–S82 27841116 10.1017/S0022215116000487 PMC4873912

[2] Department of Veterans Affairs Laryngeal Cancer Study Group, Wolf GT, Fisher SG, Hong WK, Hillman R, Spaulding M, Laramore GE, Endicott JW, McClatchey K, Henderson WG (1991) Induction chemotherapy plus radiation compared with surgery plus radiation in patients with advanced laryngeal cancer. N Engl J Med 324(24):1685–1690 2034244 10.1056/NEJM199106133242402

[3] Forastiere AA, Goepfert H, Maor M, Pajak TF, Weber R, Morrison W, Glisson B, Trotti A, Ridge JA, Chao C, Peters G, Lee DJ, Leaf A, Ensley J, Cooper J (2003) Concurrent chemotherapy and radiotherapy for organ preservation in advanced laryngeal cancer. N Engl J Med 349(22):2091–2098 14645636 10.1056/NEJMoa031317

[4] Ko HC, Harari PM, Chen S, Wieland AM, Yu M, Baschnagel AM, Kimple RJ, Witek ME (2017) Survival outcomes for patients with T3N0M0 squamous cell carcinoma of the glottic larynx. JAMA Otolaryngol Head Neck Surg 143(11):1126–1133 29049434 10.1001/jamaoto.2017.1756 PMC5710357

[5] Sanabria A, Chaves ALF, Kowalski LP, Wolf GT, Saba NF, Forastiere AA, Beitler JJ, Nibu KI, Bradford CR, Suárez C, Rodrigo JP, Strojan P, Rinaldo A, de Bree R, Haigentz M Jr, Takes RP, Ferlito A (2017) Organ preservation with chemoradiation in advanced laryngeal cancer: the problem of generalizing results from randomized controlled trials. Auris Nasus Larynx 44(1):18–25 27397024 10.1016/j.anl.2016.06.005

[6] Nocini R, Molteni G, Mattiuzzi C, Lippi G (2020) Updates on larynx cancer epidemiology. Chin J Cancer Res 32(1):18–25 32194301 10.21147/j.issn.1000-9604.2020.01.03 PMC7072014

[7] Canis M, Ihler F, Martin A, Wolff HA, Matthias C, Steiner W (2014) Results of 226 patients with T3 laryngeal carcinoma after treatment with transoral laser microsurgery. Head Neck 36(5):652–659 23596018 10.1002/hed.23338

[8] Krebs in Deutschland 2009/2010 9. Ausgabe. Robert Koch-Institut (Hrsg.) und die Gesellschaft der epidemiologischen Krebsregister in Deutschland e.V. (Hrsg.)

[9] Sikora AG, Toniolo P, DeLacure MD (2004) The changing demographics of head and neck squamous cell carcinoma in the United States. Laryngoscope 114(11):1915–1923 15510014 10.1097/01.mlg.0000147920.66486.bc

[10] Forastiere AA, Zhang Q, Weber RS, Maor MH, Goepfert H, Pajak TF, Morrison W, Glisson B, Trotti A, Ridge JA, Thorstad W, Wagner H, Ensley JF, Cooper JS (2013) Long-term results of RTOG 91-11: a comparison of three nonsurgical treatment strategies to preserve the larynx in patients with locally advanced larynx cancer. J Clin Oncol 31(7):845–852 23182993 10.1200/JCO.2012.43.6097 PMC3577950

[11] Blanchard P, Baujat B, Holostenco V, Bourredjem A, Baey C, Bourhis J, Pignon JP, MACH-CH Collaborative group (2011) Meta-analysis of chemotherapy in head and neck cancer (MACH-NC): a comprehensive analysis by tumour site. Radiother Oncol 100(1):33–40 21684027 10.1016/j.radonc.2011.05.036

[12] Pignon JP, le Maître A, Maillard E, Bourhis J (2009) MACH-NC Collaborative Group. Meta-analysis of chemotherapy in head and neck cancer (MACH-NC): an update on 93 randomised trials and 17,346 patients. Radiother Oncol 92(1):4–14 19446902 10.1016/j.radonc.2009.04.014

[13] Janoray G, Pointreau Y, Garaud P, Chapet S, Alfonsi M, Sire C, Jadaud E, Calais G (2016) Long-term results of a multicenter randomized phase III trial of induction chemotherapy with cisplatin, 5-fluorouracil, +/− docetaxel for larynx preservation. J Natl Cancer Inst. 10.1093/jnci/djv368 26681800 10.1093/jnci/djv368

[14] Dietz A et al (2016) Final results of the randomized phase II DeLOS-II trial: induction chemotherapy (IC) followed by radiotherapy (R) vs. cetuximab (E) plus IC and R for functional larynx preservation in resectable laryngeal and hypopharyngeal cancer (LHSCC). J Clin Oncol 34(15_Suppl):6025

[15] D’Ascanio L, Piazza F (2018) Survival outcomes for patients with T3N0M0 squamous cell carcinoma of the glottic larynx. JAMA Otolaryngol Head Neck Surg 144(6):542–543 29621368 10.1001/jamaoto.2018.0077

[16] Budach W, Bölke E, Kammers K, Gerber PA, Orth K, Gripp S, Matuschek C (2016) Induction chemotherapy followed by concurrent radio-chemotherapy versus concurrent radio-chemotherapy alone as treatment of locally advanced squamous cell carcinoma of the head and neck (HNSCC): a meta-analysis of randomized trials. Radiother Oncol 118(2):238–243 26589131 10.1016/j.radonc.2015.10.014

[17] Lefebvre JL, Pointreau Y, Rolland F, Alfonsi M, Baudoux A, Sire C, de Raucourt D, Malard O, Degardin M, Tuchais C, Blot E, Rives M, Reyt E, Tourani JM, Geoffrois L, Peyrade F, Guichard F, Chevalier D, Babin E, Lang P, Janot F, Calais G, Garaud P, Bardet E (2013) Induction chemotherapy followed by either chemoradiotherapy or bioradiotherapy for larynx preservation: the TREMPLIN randomized phase II study. J Clin Oncol 31(7):853–859 23341517 10.1200/JCO.2012.42.3988

[18] Bourhis J, Le Maître A, Baujat B, Audry H, Pignon JP (2007) Meta-analysis of chemotherapy in head, neck cancer collaborative group; meta-analysis of radiotherapy in carcinoma of head, neck collaborative group; meta-analysis of chemotherapy in nasopharynx carcinoma collaborative group. Individual patients’ data meta-analyses in head and neck cancer. Curr Opin Oncol 19(3):188–194 17414635 10.1097/CCO.0b013e3280f01010

[19] Lefebvre JL, Ang KK (2009) Larynx preservation consensus panel. Larynx preservation clinical trial design: key issues and recommendations—a consensus panel summary. Head Neck 31(4):429–441 19283793 10.1002/hed.21081

[20] Lefebvre JL (2006) Laryngeal preservation in head and neck cancer: multidisciplinary approach. Lancet Oncol 7(9):747–755 16945770 10.1016/S1470-2045(06)70860-9

[21] Machtay M, Moughan J, Trotti A, Garden AS, Weber RS, Cooper JS, Forastiere A, Ang KK (2008) Factors associated with severe late toxicity after concurrent chemoradiation for locally advanced head and neck cancer: an RTOG analysis. J Clin Oncol 26(21):3582–3589 18559875 10.1200/JCO.2007.14.8841 PMC4911537

[22] Graboyes EM, Zhan KY, Garrett-Mayer E, Lentsch EJ, Sharma AK, Day TA (2017) Effect of postoperative radiotherapy on survival for surgically managed pT3N0 and pT4aN0 laryngeal cancer: analysis of the National cancer data Base. Cancer 123(12):2248–2257 28182267 10.1002/cncr.30586

[23] Birchall M (2005) EaStER—early stage glottic cancer: endoscopic excision for radiotherapy: a feasibility study. National Research Register N0212194189

[24] Warner L, Chudasama J, Kelly CG, Loughran S, McKenzie K, Wight R, Dey P (2014) Radiotherapy versus open surgery versus endolaryngeal surgery (with or without laser) for early laryngeal squamous cell cancer. Cochrane Database Syst Rev 2014(12):CD002027 25503538 10.1002/14651858.CD002027.pub2 PMC6599864

[25] Ogoltsova ES (1990) Comparative evaluation of the efficacy of radiotherapy, surgery and combined treatment of stage I–II laryngeal cancer (T1–2N0M0) on the basis of cooperative studies. Vestn Oto-Rino-Laringol 3:3–7 2200195

[26] Yoo J, Lacchetti C, Hammond JA, Gilbert RW (2014) Head and Neck Cancer Disease Site Group. Role of endolaryngeal surgery (with or without laser) versus radiotherapy in the management of early (T1) glottic cancer: a systematic review. Head Neck 36(12):1807–1819 24115131 10.1002/hed.23504

[27] Higgins KM, Shah MD, Ogaick MJ, Enepekides D (2009) Treatment of early-stage glottic cancer: meta-analysis comparison of laser excision versus radiotherapy. J Otolaryngol Head Neck Surg 38(6):603–612 19958721

[28] Abdurehim Y, Hua Z, Yasin Y, Xukurhan A, Imam I, Yuqin F (2012) Transoral laser surgery versus radiotherapy: systematic review and meta-analysis for treatment options of T1a glottic cancer. Head Neck 34(1):23–33 21374753 10.1002/hed.21686

[29] Feng Y, Wang B, Wen S (2011) Laser surgery versus radiotherapy for T1–T2N0 glottic cancer: a meta-analysis. ORL J Oto-Rhino-Laryngol Relat Spec 73(6):336–342 10.1159/000327097 22005723

[30] Wei WI (2002) The dilemma of treating hypopharyngeal carcinoma: more or less: Hayes Martin Lecture. Arch Otolaryngol Head Neck Surg 128(3):229–232 11886334 10.1001/archotol.128.3.229

[31] Mimica X, Hanson M, Patel SG, McGill M, McBride S, Lee N, Dunn LA, Cracchiolo JR, Shah JP, Wong RJ, Ganly I, Cohen MA (2019) Salvage surgery for recurrent larynx cancer. Head Neck 41(11):3906–3915 31433540 10.1002/hed.25925 PMC7485603

[32] Tupper C (1965) Radical Wertheim as a salvage procedure. Patients with recurrence following initial definitive radiotherapy. Am J Obstet Gynecol 91:364–368 14258263 10.1016/0002-9378(65)90251-6

[33] Sanabria A, Kowalski LP, Shaha AR, Silver CE, Werner JA, Mandapathil M, Takes RP, Strojan P, Rinaldo A, Ferlito A (2014) Salvage surgery for head and neck cancer: a plea for better definitions. Eur Arch Otorhinolaryngol 271(6):1347–1350 24532050 10.1007/s00405-014-2924-7

