# Supplementary material for: Treatment regimens for laryngeal and hypopharyngeal squamous cell carcinoma: a “real life” multicenter study of 2307 patients
Source: Eur Arch Otorhinolaryngol. 2024 Oct 22;282(12):6609–21. doi: 10.1007/s00405-024-08990-6 (PMC12680893; doi:10.1007/s00405-024-08990-6)
Supplement: Supplementary file 1 — Supplementary file1 (DOCX 45 KB) [file 405_2024_8990_MOESM1_ESM.docx]

**Supplement 1**

|  | **T1/2 N0/+ M0 supraglottic cancer** | | | **T3/4 N0/+ M0 supraglottic cancer** | | | **T1-4 N0/+ M0 subglottic cancer** | | |
| --- | --- | --- | --- | --- | --- | --- | --- | --- | --- |
|  | **LpreS** | **pC/RT** | **p-value** | **L(P)E** | **pC/RT** | **p-value** | **Surgery** | **pC/RT** | **p-value** |
| **N** | 146 | 33 |  | 83 | 71 |  | 16 | 11 |  |
| **Age (years)** |  |  | <0.0001 |  |  | 0.42 |  |  | 0.33 |
| Median | 60 | 73 |  | 60 | 65 |  | 67 | 64 |  |
| Mean ± SD | 60±9 | 71±9 |  | 61±9 | 62±11 |  | 63±13 | 69±13 |  |
| Range | 38-88 | 34-89 |  | 41-84 | 39-89 |  | 43-79 | 51-88 |  |
| **Sex, n (%)** |  |  | 0.13 |  |  | 0.02 |  |  | 0.79 |
| Male | 116 (80) | 30 (91) |  | 73 (88) | 52 (73) |  | 15 (94) | 10 (91) |  |
| Female | 30 (20) | 3 (9) |  | 10 (12) | 19 (27) |  | 1 (6) | 1 (9) |  |
| **T status, n (%)** |  |  | 0.08 |  |  | 0.97 |  |  | 0.23 |
| T1 | 64 (44) | 9 (27) |  |  |  |  | 4 (25) | 0 |  |
| T2 | 82 (56) | 24 (73) |  |  |  |  | 2 (12) | 3 (27) |  |
| T3 |  |  |  | 47 (57) | 40 (56) |  | 3 (19) | 1 (9) |  |
| T4 |  |  |  | 36 (43) | 31 (44) |  | 7 (44) | 7 (64) |  |
| **N status, n (%)** |  |  | 0.002 |  |  | <0.0001 |  |  | 0.78 |
| N0 | 88 (60) | 13 (39) |  | 41 (49) | 17 (24) |  | 12 (75) | 7 (64) |  |
| N1 | 26 (18) | 7 (21) |  | 18 (22) | 5 (7) |  | 1 (6) | 1 (9) |  |
| N2a | 8 (6) | 1 (3) |  | 1 (1) | 5 (7) |  | 0 | 1 (9) |  |
| N2b | 17 (12) | 4 (12) |  | 7 (8) | 21 (30) |  | 1 (6) | 1 (9) |  |
| N2c | 7 (5) | 6 (18) |  | 16 (19) | 18 (25) |  | 2 (12) | 1 (9) |  |
| N3 | 0 | 2 (6) |  | 0 | 5 (7) |  | 0 | 0 |  |
| **Grading, n (%)** |  |  | 0.65 |  |  | 0.009 |  |  | 0.24 |
| G1 | 4 (3) | 1 (3) |  | 2 (2) | 0 |  | 0 | 0 |  |
| G2 | 90 (62) | 18 (55) |  | 66 (80) | 42 (59) |  | 11 (69) | 9 (82) |  |
| G3 | 39 (27) | 10 (30) |  | 15 (18) | 23 (32) |  | 2 (12) | 1 (9) |  |
| G4 | 0 | 0 |  | 0 | 0 |  | 2 (12) | 0 |  |
| Gx | 13 (9) | 4 (12) |  | 0 | 6 (9) |  | 1 (6) | 1 (9) |  |
| **R status, n (%)** |  |  |  |  |  |  |  |  |  |
| R0 | 124 (85) |  |  | 78 (94) |  |  | 15 (94) |  |  |
| R1 | 6 (4) |  |  | 5 (6) |  |  | 0 |  |  |
| R2 | 0 |  |  | 0 |  |  | 0 |  |  |
| Rx | 16 (11) |  |  | 0 |  |  | 1 (6) |  |  |
| **Therapy, n (%)** |  |  |  |  |  |  |  |  |  |
| Surgery |  |  |  |  |  |  |  |  |  |
| - transoral | 113 (77) |  |  |  |  |  | 3 (19) |  |  |
| - part. LE, horizontal | 15 (10) |  |  |  |  |  | 0 |  |  |
| - part. LE, vertical | 9 (6) |  |  |  |  |  | 1 (6) |  |  |
| - Lateral pharyngotomy | 9 (6) |  |  |  |  |  | 0 |  |  |
| - LE |  |  |  | 70 (84) |  |  | 12 (75) |  |  |
| - LPE |  |  |  | 13 (16) |  |  | 0 |  |  |
| Neck dissection |  |  |  |  |  |  |  |  |  |
| - None | 41 (28) | 33 (100) |  | 0 | 69 (97) |  | 4 (25) | 11 (100) |  |
| - Ipsilateral | 27 (19) | 0 |  | 8 (10) | 2 (3) |  | 2 (12) | 0 |  |
| - Bilateral | 78 (53) | 0 |  | 75 (90) | 0 |  | 10 (63) | 0 |  |
| Adjuvant treatment |  |  |  |  |  |  |  |  |  |
| - RT | 61 (42) |  |  | 47 (57) |  |  | 7 (41) |  |  |
| - CRT | 23 (16) |  |  | 11 (13) |  |  | 2 (12) |  |  |
| Primary cons. treatment |  |  |  |  |  |  |  |  |  |
| - RT |  | 14 (42) |  |  | 18 (25) |  |  | 7 (64) |  |
| - CRT |  | 19 (58) |  |  | 53 (75) |  |  | 4 (36) |  |

**Supplement 2**

|  | **T1/2 N0 M0 glottic cancer** | | | **T3 N0 M0 glottic cancer** | | | **T4 N0/+ M0 glottic cancer** | | |
| --- | --- | --- | --- | --- | --- | --- | --- | --- | --- |
|  | **LpreS** | **pC/RT** | **p-value** | **L(P)E** | **pC/RT** | **p-value** | **L(P)E** | **pC/RT** | **p-value** |
| **N** | 601 | 104 |  | 39 | 37 |  | 96 | 21 |  |
| **Age (years)** |  |  | 0.12 |  |  | 0.001 |  |  | 0.32 |
| Median | 64 | 68 |  | 64 | 74 |  | 62 | 66 |  |
| Mean ± SD | 64±10 | 66±12 |  | 64±9 | 72±11 |  | 64±9 | 66±9 |  |
| Range | 28-89 | 34-89 |  | 38-83 | 56-89 |  | 39-82 | 51-80 |  |
| **Sex, n (%)** |  |  | 0.66 |  |  | 0.44 |  |  | 0.69 |
| Male | 551 (92) | 94 (90) |  | 35 (90) | 35 (95) |  | 89 (93) | 20 (95) |  |
| Female | 50 (8) | 10 (10) |  | 4 (10) | 2 (5) |  | 7 (7) | 1 (5) |  |
| **T status, n (%)** |  |  | <0.0001 |  |  |  |  |  |  |
| T1 | 472 (78) | 59 (57) |  |  |  |  |  |  |  |
| T2 | 129 (22) | 45 (43) |  |  |  |  |  |  |  |
| T3 |  |  |  | 39 (100) | 37 (100) |  |  |  |  |
| T4 |  |  |  |  |  |  | 96 (100) | 21 (100) |  |
| **N status, n (%)** |  |  |  |  |  |  |  |  | 0.02 |
| N0 | 601 (100) | 104 (100) |  | 39 (100) | 37 (100) |  | 63 (66) | 7 (33) |  |
| N1 |  |  |  |  |  |  | 12 (13) | 6 (29) |  |
| N2a |  |  |  |  |  |  | 2 (2) | 1 (5) |  |
| N2b |  |  |  |  |  |  | 12 (13) | 1 (5) |  |
| N2c |  |  |  |  |  |  | 7 (7) | 6 (29) |  |
| N3 |  |  |  |  |  |  | 0 |  |  |
| **Grading, n (%)** |  |  | 0.58 |  |  | 0.99 |  |  | 0.56 |
| G1 | 115 (19) | 22 (21) |  | 6 (15) | 0 |  | 5 (5) | 0 |  |
| G2 | 421 (70) | 71 (68) |  | 27 (69) | 35 (95) |  | 73 (76) | 17 (81) |  |
| G3 | 58 (10) | 9 (9) |  | 6 (15) | 0 |  | 17 (18) | 4 (19) |  |
| G4 | 0 | 0 |  | 0 | 0 |  | 0 | 0 |  |
| Gx | 7 (1) | 2 (2) |  | 0 | 2 (5) |  | 1 (1) | 0 |  |
| **R status, n (%)** |  |  |  |  |  |  |  |  |  |
| R0 | 500 (83) |  |  | 38 (97) |  |  | 93 (97) |  |  |
| R1 | 25 (4) |  |  | 1 (3) |  |  | 2 (2) |  |  |
| R2 | 0 |  |  | 0 |  |  | 0 |  |  |
| Rx | 76 (13) |  |  | 0 |  |  | 1 (1) |  |  |
| **Therapy, n (%)** |  |  |  |  |  |  |  |  |  |
| Surgery |  |  |  |  |  |  |  |  |  |
| - transoral | 500 (83) |  |  |  |  |  |  |  |  |
| - part. LE, horizontal | 0 |  |  |  |  |  |  |  |  |
| - part. LE, vertical | 99 (17) |  |  |  |  |  |  |  |  |
| - Lateral pharyngotomy | 3 |  |  |  |  |  |  |  |  |
| - LE |  |  |  | 37 (95) |  |  | 84 (88) |  |  |
| - LPE |  |  |  | 2 (5) |  |  | 12 (12) |  |  |
| Neck dissection |  |  |  |  |  |  |  |  |  |
| - None | 568 (95) | 104 (100) |  | 4 (10) |  |  | 0 |  |  |
| - Ipsilateral | 21 (3) | 0 |  | 4 (10) |  |  | 9 (9) |  |  |
| - Bilateral | 12 (2) | 0 |  | 31 (80) |  |  | 87 (91) |  |  |
| Adjuvant treatment |  |  |  |  |  |  |  |  |  |
| - RT | 20 (3) |  |  | 13 (33) |  |  | 49 (51) |  |  |
| - CRT | 3 |  |  | 2 (5) |  |  | 22 (23) |  |  |
| Primary cons. treatment |  |  |  |  |  |  |  |  |  |
| - RT |  | 82 (79) |  |  | 15 (41) |  |  | 4 (19) |  |
| - CRT |  | 22 (21) |  |  | 23 (62) |  |  | 17 (81) |  |

**Supplement 3**

|  | **T1/2 N0/+ M0 hypopharyngeal cancer** | | | **T1/2 N0/+ M0 hypopharyngeal cancer** | | | **T3/4 N0/+ M0 hypopharyngeal cancer** | | | |
| --- | --- | --- | --- | --- | --- | --- | --- | --- | --- | --- |
|  | **LpreS** | **pC/RT** | **p-value** | **LpreS** | **L(P)E** |  | **L(P)E** | **pC/RT** | **p-value** |  |
| **N** | 165 | 83 |  | 165 | 32 |  | 130 | 275 |  |  |
| **Age (years)** |  |  | 0.17 |  |  | 0.78 |  |  | 0.42 |  |
| Median | 60 | 61 |  | 60 | 60 |  | 61 | 58 |  |  |
| Mean ± SD | 59±9 | 61±7 |  | 59±9 | 60±7 |  | 60±10 | 60±10 |  |  |
| Range | 43-82 | 46-78 |  | 43-82 | 44-75 |  | 33-82 | 41-84 |  |  |
| **Sex, n (%)** |  |  | 0.61 |  |  | 0.85 |  |  | 0.21 |  |
| Male | 137 (83) | 71 (86) |  | 137 (83) | 27 (84) |  | 119 (92) | 240 (87) |  |  |
| Female | 28 (17) | 12 (14) |  | 28 (17) | 5 (16) |  | 11 (8) | 35 (13) |  |  |
| **T status, n (%)** |  |  | <0.0001 |  |  | <0.0001 |  |  | 0.38 |  |
| T1 | 70 (42) | 9 (11) |  | 70 (42) | 1 (3) |  |  |  |  |  |
| T2 | 98 (58) | 74 (89) |  | 98 (58) | 31 (97) |  |  |  |  |  |
| T3 |  |  |  |  |  |  | 58 (45) | 100 (40) |  |  |
| T4 |  |  |  |  |  |  | 72 (55) | 175 (60) |  |  |
| **N status, n (%)** |  |  | <0.0001 |  |  | 0.57 |  |  | 0.053 |  |
| N0 | 50 (30) | 14 (17) |  | 50 (30) | 11 (34) |  | 31 (24) | 44 (16) |  |  |
| N1 | 37 (22) | 11 (13) |  | 37 (22) | 3 (9) |  | 14 (11) | 30 (11) |  |  |
| N2a | 11 (7) | 4 (5) |  | 11 (7) | 2 (6) |  | 8 (6) | 17 (6) |  |  |
| N2b | 54 (33) | 18 (22) |  | 54 (33) | 12 (38) |  | 39 (30) | 91 (33) |  |  |
| N2c | 4 (2) | 26 (31) |  | 4 (2) | 3 (9) |  | 36 (28) | 76 (28) |  |  |
| N3 | 9 (6) | 10 (12) |  | 9 (6) | 1 (3) |  | 2 (2) | 17 (6) |  |  |
| **Grading, n (%)** |  |  | 0.03 |  |  | 0.1 |  |  | 0.60 |  |
| G1 | 4 (2) | 2 (2) |  | 4 (2) | 0 |  | 0 | 9 (3) |  |  |
| G2 | 98 (59) | 39 (47) |  | 98 (59) | 17 (53) |  | 82 (63) | 154 (56) |  |  |
| G3 | 50 (30) | 35 (42) |  | 50 (30) | 15 (47) |  | 46 (35) | 91 (33) |  |  |
| G4 | 0 | 2 (2) |  | 0 | 0 |  | 0 | 1 |  |  |
| Gx | 13 (8) | 5 (6) |  | 13 (8) | 0 |  | 2 (2) | 20 (7) |  |  |
| **R status, n (%)** |  |  |  |  |  | 0.025 |  |  |  |  |
| R0 | 128 (78) |  |  | 128 (78) | 29 (91) |  | 102 (79) |  |  |  |
| R1 | 17 (10) |  |  | 17 (10) | 2 (6) |  | 25 (19) |  |  |  |
| R2 | 1 (1) |  |  | 1 (1) | 0 |  | 2 (2) |  |  |  |
| Rx | 19 (12) |  |  | 19 (12) | 1 (4) |  | 1 (1) |  |  |  |
| **Therapy, n (%)** |  |  |  |  |  |  |  |  |  |  |
| Surgery |  |  |  |  |  |  |  |  |  |  |
| - transoral | 110 (67) |  |  | 110 (67) |  |  |  |  |  |  |
| - part. LE, horizontal | 17 (10) |  |  | 17 (10) |  |  |  |  |  |  |
| - part. LE, vertical | 0 |  |  | 0 |  |  |  |  |  |  |
| - Lateral pharyngotomy | 38 (23) |  |  | 38 (23) |  |  |  |  |  |  |
| - LE |  |  |  |  | 15 (47) |  |  |  |  |  |
| - LPE |  |  |  |  | 17 (53) |  | 130 (100) |  |  |  |
| Neck dissection |  |  |  |  |  | <0.0001 |  |  |  |  |
| - None | 19 (12) | 79 (95) |  | 19 (12) | 0 |  | 3 (2) | 272 (99) |  |  |
| - Ipsilateral | 83 (50) | 4 (5) |  | 83 (50) | 2 (6) |  | 20 (15) | 3 (1) |  |  |
| - Bilateral | 63 (38) | 0 |  | 63 (38) | 30 (94) |  | 107 (82) | 0 |  |  |
| Adjuvant treatment |  |  |  |  |  | 0.13 |  |  |  |  |
| - RT | 65 (39) |  |  | 65 (39) | 6 (19) |  | 49 (38) |  |  |  |
| - CRT | 62 (38) |  |  | 62 (38) | 19 (59) |  | 61 (47) |  |  |  |
| Primary cons. treatment |  |  |  |  |  |  |  |  |  |  |
| - RT |  | 18 (22) |  |  |  |  |  | 26 (10) |  |  |
| - CRT |  | 65 (78) |  |  |  |  |  | 249 (90) |  |  |

**Supplement 4**

|  | **2004** | **2005** | **2006** | **2007** | **2008** | **2009** | **2010** | **2011** | **2012** | **2013** | **2014** | **R²** | **p-value** |
| --- | --- | --- | --- | --- | --- | --- | --- | --- | --- | --- | --- | --- | --- |
| **n** | 55 | 69 | 66 | 69 | 70 | 74 | 71 | 66 | 70 | 106 | 67 |  |  |
| **Age (years)** |  |  |  |  |  |  |  |  |  |  |  | 0.02 | <0.0001 |
| Median | 57 | 61 | 61 | 61 | 61 | 61 | 65 | 60 | 65 | 63 | 63 |  |  |
| Mean ± SD | 57±10 | 60±10 | 60±11 | 60±9 | 61±10 | 61±10 | 64±10 | 60±9 | 65±9 | 63±9 | 63±10 |  |  |
| Range | 37-78 | 42-80 | 38-90 | 40-80 | 41-81 | 39-84 | 45-83 | 45-83 | 41-87 | 33-80 | 33-83 |  |  |
| **Sex, n (%)** |  |  |  |  |  |  |  |  |  |  |  | 0.001 | 0.36 |
| Male | 51 (93) | 59 (86) | 60 (91) | 62 (90) | 66 (94) | 67 (91) | 61 (86) | 57 (86) | 68 (97) | 91 (86) | 57 (85) |  |  |
| Female | 4 (7) | 10 (14) | 6 (9) | 7 (10) | 4 (6) | 7 (9) | 9 (14) | 9 (14) | 2 (3) | 15 (14) | 10 (15) |  |  |
| **Location, n (%)** |  |  |  |  |  |  |  |  |  |  |  | 0.03 | 0.024 |
| Supraglottis | 16 (29) | 20 (29) | 17 (26) | 14 (20) | 20 (29) | 12 (16) | 8 (11) | 6 (9) | 9 (13) | 26 (25) | 15 (22) |  |  |
| Glottis | 19 (35) | 23 (33) | 22 (33) | 31 (45) | 19 (27) | 33 (45) | 22 (31) | 23 (35) | 29 (41) | 38 (36) | 28 (42) |  |  |
| Subglottis | 6 (11) | 3 (4) | 4 (6) | 0 | 1 (1) | 2 (3) | 3 (4) | 3 (5) | 0 | 2 (2) | 3 (5) |  |  |
| Hypopharynx | 8 (15) | 16 (23) | 15 (23) | 10 (15) | 20 (29) | 18 (24) | 29 (41) | 22 (33) | 18 (26) | 23 (22) | 15 (22) |  |  |
| Oropharynx | 6 (11) | 7 (10) | 7 (11) | 14 (20) | 10 (14) | 9 (12) | 9 (13) | 12 (18) | 12 (17) | 17 (16) | 6 (9) |  |  |
| Other | 0 | 0 | 1 (2) | 0 | 0 | 0 | 0 | 0 | 2 (3) | 0 | 0 |  |  |
| **T status, n (%)** |  |  |  |  |  |  |  |  |  |  |  | 0.001 | 0.64 |
| T1 | 1 (2) | 0 | 1 (2) | 2 (3) | 1 (1) | 0 | 1 (1) | 1 (2) | 1 (1) | 3 (3) | 2 (3) |  |  |
| T2 | 6 (11) | 3 (4) | 6 (9) | 7 (10) | 5 (7) | 14 (19) | 16 (23) | 16 (24) | 8 (11) | 11 (10) | 5 (8) |  |  |
| T3 | 22 (40) | 32 (46) | 32 (49) | 27 (39) | 22 (31) | 22 (30) | 26 (37) | 16 (24) | 21 (30) | 25 (24) | 13 (19) |  |  |
| T4 | 26 (47) | 34 (49) | 27 (41) | 33 (48) | 42 (60) | 38 (51) | 28 (40) | 33 (50) | 40 (57) | 67 (63) | 47 (70) |  |  |
| **N status, n (%)** |  |  |  |  |  |  |  |  |  |  |  | 0.008 | 0.27 |
| N0 | 27 (49) | 36 (52) | 31 (47) | 33 (48) | 27 (39) | 39 (53) | 39 (55) | 33 (50) | 30 (43) | 57 (54) | 35 (52) |  |  |
| N1 | 12 (22) | 8 (12) | 7 (11) | 14 (20) | 14 (20) | 5 (7) | 10 (14) | 10 (15) | 13 (19) | 15 (14) | 6 (9) |  |  |
| N2a | 5 (9) | 2 (3) | 2 (3) | 2 (3) | 4 (6) | 3 (4) | 1 (1) | 2 (3) | 3 (4) | 4 (4) | 1 (2) |  |  |
| N2b | 9 (16) | 12 (17) | 11 (17) | 12 (17) | 12 (17) | 14 (19) | 10 (14) | 12 (18) | 11 (16) | 17 (16) | 15 (22) |  |  |
| N2c | 2 (4) | 11 (16) | 15 (23) | 8 (12) | 11 (16) | 12 (16) | 9 (13) | 9 (14) | 13 (19) | 11 (10) | 10 (15) |  |  |
| N3 | 0 | 0 | 0 | 0 | 2 (3) | 1 (1) | 2 (3) | 0 | 0 | 2 (2) | 0 |  |  |
| **M status, n (%)** |  |  |  |  |  |  |  |  |  |  |  | 0.003 | 0.53 |
| M0 | 53 (96) | 66 (96) | 65 (99) | 67 (97) | 66 (94) | 71 (96) | 70 (99) | 66 (100) | 67 (96) | 101 (95) | 65 (97) |  |  |
| M1 | 2 (4) | 3 (4) | 1 (1) | 2 (3) | 4 (6) | 3 (4) | 1 (1) | 0 | 3 (4) | 5 (5) | 2 (3) |  |  |
| **Grading, n (%)** |  |  |  |  |  |  |  |  |  |  |  | 0.003 | 0.49 |
| G1 | 5 (9) | 1 (1) | 2 (3) | 4 (6) | 1 (1) | 2 (3) | 3 (4) | 3 (5) | 2 (3) | 3 (3) | 0 |  |  |
| G2 | 31 (56) | 42 (61) | 38 (58) | 36 (52) | 38 (54) | 48 (65) | 44 (62) | 46 (70) | 36 (51) | 66 (62) | 52 (78) |  |  |
| G3 | 18 (33) | 26 (38) | 25 (38) | 29 (42) | 31 (44) | 24 (32) | 22 (31) | 17 (26) | 32 (46) | 37 (35) | 15 (22) |  |  |
| G4 | 1 (2) | 0 | 1 (2) | 0 | 0 | 0 | 2 (3) | 0 | 0 | 0 | 0 |  |  |
| **R status, n (%)** |  |  |  |  |  |  |  |  |  |  |  | 0.03 | 0.11 |
| R0 | 54 (98) | 56 (81) | 61 (92) | 64 (93) | 63 (90) | 73 (99) | 58 (82) | 60 (91) | 61 (87) | 94 (89) | 62 (93) |  |  |
| R1 | 1 (2) | 11 (16) | 3 (5) | 5 (7) | 6 (9) | 1 (1) | 10 (14) | 5 (8) | 8 (11) | 10 (9) | 3 (5) |  |  |
| R2 | 0 | 2 (3) | 1 (2) | 0 | 0 | 0 | 2 (3) | 1 (2) | 0 | 0 | 1 (2) |  |  |
| Rx | 0 | 0 | 1 (2) | 0 | 1 (1) | 0 | 1 (1) | 0 | 1 (1) | 2 (2) | 1 (2) |  |  |
| **Indication for L(P)E** |  |  |  |  |  |  |  |  |  |  |  | 0.01 | 0.005 |
| rT+ | 3 (5) | 12 (17) | 11 (17) | 11 (16) | 23 (33) | 17 (23) | 6 (10) | 11 (17) | 19 (27) | 27 (25) | 18 (27) |  |  |
| First pass | 52 (95) | 57 (83) | 55 (83) | 58 (84) | 47 (67) | 57 (77) | 64 (90) | 55 (83) | 51 (73) | 79 (75) | 49 (73) |  |  |
| **Surgery, n (%)** |  |  |  |  |  |  |  |  |  |  |  | <0.0001 | 0.95 |
| LE | 49 (89) | 58 (84) | 49 (74) | 53 (77) | 47 (67) | 56 (76) | 42 (59) | 38 (58) | 48 (69) | 66 (62) | 39 (58) |  |  |
| LPE | 6 (11) | 11 (16) | 17 (26) | 16 (23) | 23 (33) | 18 (24) | 29 (41) | 28 (42) | 22 (31) | 40 (38) | 28 (42) |  |  |
| **Neck dissection, n (%)** |  |  |  |  |  |  |  |  |  |  |  | 0.01 | 0.15 |
| None | 0 | 4 (9) | 1 (2) | 3 (4) | 2 (3) | 1 (1) | 1 (1) | 2 (3) | 5 (7) | 5 (5) | 4 (6) |  |  |
| Ipsi-lateral | 5 (9) | 13 (19) | 9 (14) | 6 (9) | 7 (10) | 4 (5) | 7 (10) | 1 (2) | 5 (7) | 2 (2) | 6 (9) |  |  |
| Bi-lateral | 50 (91) | 52 (75) | 56 (85) | 60 (87) | 31 (87) | 69 (94) | 63 (89) | 63 (96) | 60 (86) | 99 (93) | 57 (85) |  |  |
| **Adjuvant Therapy** |  |  |  |  |  |  |  |  |  |  |  | 0.005 | 0.37 |
| None | 18 (33) | 14 (20) | 23 (35) | 12 (17) | 26 (37) | 22 (30) | 22 (31) | 10 (15) | 19 (27) | 34 (32) | 23 (34) |  |  |
| RT | 26 (47) | 29 (42) | 24 (36) | 25 (36) | 20 (29) | 31 (42) | 21 (30) | 22 (33) | 26 (37) | 36 (34) | 21 (31) |  |  |
| CRT | 11 (20) | 26 (38) | 19 (29) | 32 (46) | 24 (34) | 21 (28) | 28 (39) | 34 (52) | 25 (36) | 36 (34) | 23 (34) |  |  |
| **Postoperative complication, n (%)** |  |  |  |  |  |  |  |  |  |  |  | 0.003 | 0.14 |
| None | 50 (90) | 57 (83) | 58 (88) | 57 (83) | 58 (83) | 66 (89) | 59 (83) | 52 (79) | 60 (86) | 83 (78) | 57 (85) |  |  |
| Lung embolism | 0 | 2 (3) | 2 (3) | 1 (1) | 0 | 0 | 2 (3) | 0 | 0 | 1 (1) | 0 |  |  |
| Cardiac infarction | 0 | 0 | 0 | 0 | 0 | 2 (3) | 0 | 1 (2) | 0 | 0 | 0 |  |  |
| Cardiac arrhythmia | 0 | 0 | 0 | 0 | 0 | 0 | 1 (1) | 0 | 0 | 3 (3) | 2 (3) |  |  |
| Cerebrovascular accident | 1 (2) | 2 (3) | 0 | 0 | 0 | 0 | 0 | 0 | 1 (1) | 2 (2) | 0 |  |  |
| Hormone crisis | 0 | 0 | 0 | 0 | 0 | 0 | 0 | 0 | 0 | 0 | 0 |  |  |
| Vessel blow-out | 0 | 0 | 3 (5) | 0 | 0 | 0 | 0 | 1 (2) | 0 | 1 (1) | 1 (2) |  |  |
| Pharyngo-cutaneous fistula | 3 (6) | 7 (10) | 3 (5) | 11 (16) | 12 (17) | 4 (5) | 9 (13) | 11 (17) | 9 (13) | 13 (12) | 6 (9) |  |  |
| Multiple | 1 (2) | 1 (1) | 0 | 0 | 0 | 1 (1) | 0 | 1 (2) | 0 | 3 (3) | 1 (2) |  |  |
| **Hospitalization time, n (%)** |  |  |  |  |  |  |  |  |  |  |  | <0.0001 | 0.89 |
| Median | 20 | 21 | 21 | 21 | 25 | 19 | 20 | 21 | 22 | 19 | 21 |  |  |
| Mean ± SD | 28±49 | 28±26 | 27±18 | 25±15 | 32±20 | 26±21 | 26±16 | 37±52 | 24±10 | 27±24 | 30±31 |  |  |
| Range | 4-377 | 8-142 | 11-101 | 12-103 | 11-102 | 4-127 | 3-88 | 10-38 | 10-46 | 8-152 | 7-229 |  |  |
